# Effective control of the emerging PEDV G2-c variant with an inactivated autogenous vaccine

**DOI:** 10.3389/fvets.2025.1697499

**Published:** 2025-10-14

**Authors:** Huan Xu, Haichao Wu, Juanjuan Min, Ningning Fu, Ying Shi, Ping Zhou, Mingwei Wang, Ang Shi, Yushuang Zhou, Jinhua Chen, Yuli Hu, Wen Sun, Taotao Yang

**Affiliations:** ^1^Sinopharm Animal Health Corporation Ltd., Wuhan, Hubei, China; ^2^School of Life Sciences and Environmental Resources, Yichun University, Yichun, Jiangxi, China

**Keywords:** porcine epidemic diarrhea virus, autogenous vaccine, control, G2-c variant, herd immunity

## Abstract

**Background:**

Porcine epidemic diarrhea virus (PEDV) is one of the most significant pathogens threatening the swine industry, causing severe economic losses in China. PEDV exhibits a high mutation rate, which may compromise the protective efficacy of currently available commercial vaccines.

**Results:**

This report describes a PED outbreak occurring from February to April 2025 in a southern Hunan swine farm, resulting in approximately 3,000 neonatal piglet deaths within 1 week of birth. We isolated a PEDV strain (XWF/2025) from infected piglet intestinal contents, which phylogenetic analysis classified within the G2-c subgroup. An inactivated autogenous vaccine using intestinal tissues from infected piglets was rapidly developed, evaluated and administered to all sows in the affected unit. Remarkably, piglet mortality rates decreased significantly within 2 weeks post-vaccination. Serological analysis demonstrated substantial increases in anti-PEDV neutralizing antibody titers following vaccination compared to pre-immunization levels. This represents a successful application of an autogenous inactivated vaccine for emergency PED control, providing a safe and effective approach.

**Conclusions:**

Our findings offer valuable insights for combating PED outbreaks through rapid, farm-specific vaccine development.

## Introduction

Porcine epidemic diarrhea virus (PEDV), a member of the *Alphacoronavirus* genus (*Coronaviridae* family), is the causative agent of porcine epidemic diarrhea (PED), a highly contagious enteric disease characterized by acute vomiting, profuse watery diarrhea, and severe dehydration (1). While affecting swine of all ages, the disease manifests with particularly devastating consequences in neonatal piglets, where mortality rates can approach 100% (2). In China's intensive swine production systems, recurrent PED outbreaks since 2010 have resulted in substantial economic losses, prompting urgent needs for effective control measures (1).

Molecular epidemiological surveillance has identified two major PEDV genogroups circulating in China: the classical G1 strains (e.g., CV777) exhibiting moderate pathogenicity, and the emergent G2 variants demonstrating enhanced virulence (3–8). The G2 genotype has further diversified into three distinct subgroups: G2-a (AH2012-like), G2-b (AJ1102-like), and G2-c (TJbc2023-like) (9). This strain diversity stems primarily from the error-prone replication of PEDV's 28 kb positive-sense RNA genome, particularly in the spike (S) glycoprotein gene (10).

The S protein, comprising approximately 1,385 amino acids, serves as the principal determinant of viral pathogenicity and immunogenicity (11–14). Its receptor-binding and fusion domains (including the critical COE antigenic region, aa 499–638) are frequent sites of mutations that enable immune evasion (15). Current vaccination strategies in China employ either: (i) inactivated whole-virus preparations (G1 or G2a strains) with demonstrated safety but limited immunogenicity, or (ii) live-attenuated vaccines offering superior mucosal immunity but carrying theoretical risks of reversion to virulence (16). The rapid antigenic drift of circulating strains frequently renders these vaccines ineffective, creating an urgent need for alternative approaches (8).

Here, we present a successful outbreak containment strategy combining rapid diagnostic identification of a G2-c variant strain and development of a farm-specific inactivated autogenous vaccine derived from intestinal tissues of PEDV infected piglets. Our approach addresses the critical gap between vaccine development timelines and emergent strain evolution, providing a model for responsive PED management.

## Materials and methods

### Clinical presentation and characteristics of the case farm

From February 10, 2025, a swine farm in southern Hunan, China, with approximately 4,600 sows, experienced an acute clinical signs exclusively in piglets under 7 days of age. The affected piglets presented with watery diarrhea and vomiting, leading to rapid dehydration and death, with a 100% mortality rate. In total, approximately 3,000 piglets succumbed to the illness, resulting in a financial loss of at least 1,800,000 yuan.

The farm operates with three production lines with a continuous production model, and selling nursery pigs. Routine immunization programs for the sows and gilts include vaccinations against classical swine fever, porcine reproductive and respiratory syndrome, pseudorabies, foot-and-mouth disease, Japanese encephalitis, and parvovirus. Specifically, sows are administered an attenuated live PED vaccine (CV777 strain) 5 weeks prior to farrowing, along with inactivated PED vaccines (CV777 strain) at 4 and 1 week(s) before delivery.

### Diarrheic pathogen examination

Intestinal samples from three affected piglets underwent comprehensive diagnostic testing of potential diarrheic pathogens. Nucleic acids were extracted using an automated extraction system (TIANLONG, China) and analyzed by RT-qPCR (Takara, China) for detection of porcine epidemic diarrhea virus (PEDV), porcine deltacoronavirus (PDCoV), transmissible gastroenteritis virus (TGEV), rotavirus (PoRV), and porcine bocavirus (PBoV). PCR amplification was carried out on a Gentier 96R (TIANLONG, China). The thermal profile consisted of an initial denaturation at 95 °C for 10 s. Forty cycles of amplification were then performed, each comprising denaturation at 95 °C for 5 s and combined annealing/extension at 60 °C for 30 s. The cycle threshold (Ct) was automatically determined by the instrument software, and samples without a detectable Ct value after 40 cycles were classified as negative.

### Virus isolation and characterization

Intestinal homogenates from diarrheic piglets were filtered (0.22 μm) and inoculated onto Vero cell monolayers maintained in DMEM supplemented with 5 μg/mL trypsin (Solarbio, China). Following three freeze-thaw cycles and centrifugation (4,000 rpm, 5 min), supernatants were serially passaged three times in fresh Vero cells. Viral stocks were stored at −80 °C for subsequent analysis.

Viral titers were determined by 50% tissue culture infective dose (TCID_50_) assay in 96-well Vero cell cultures. Tenfold serial dilutions were inoculated in octuplicate (100 μL/well) and incubated at 37 °C with 5% CO_2_ for 4 days. Cytopathic effects were microscopically evaluated, and titers were calculated using the Reed-Muench method.

Vero cells infected at MOI 0.01 were fixed (4% paraformaldehyde, 15 min) and permeabilized (0.2% Triton X-100, 10 min) at 24 h post-infection (hpi). After incubation with PEDV N-specific monoclonal antibodies (Qianxun Biology, China) and FITC-conjugated goat anti-mice IgG (1:800, Thermo Scientific, USA), fluorescence was visualized (Zeiss Axio Observer, Germany).

PEDV-infected Vero cell supernatants were concentrated by centrifugation (4,000 rpm, 5 min), filtered (0.22 μm), and negatively stained with 2% phosphotungstic acid for TEM examination (Hitachi HC-1, Japan).

### Genomic sequencing and analysis

Viral RNA was extracted from supernatants and reverse transcribed (RevertAid First Strand cDNA Synthesis Kit, Thermo Scientific). The complete genome of PEDV was amplified using Pfu DNA polymerase (Vazyme, China) with previously described primers (17). The complete S gene sequences of PEDVs were aligned using the MegAlign module of DNAStar. A maximum-likelihood phylogenetic tree was constructed with MEGA 6.06 under the Tamura-Nei model, incorporating a discrete gamma distribution for rate heterogeneity. Branch support was assessed with 1,000 non-parametric bootstrap replicates.

### Vaccine preparation, quality test, and immunization protocol

Intestinal tissues from diarrheic piglets confirmed as PEDV infection were physically ground with saline solution at a ratio of 1:2 (100 g of tissue was added to 200 ml of saline solution). The homogenates then centrifuged at 6,000 rpm for 15 min. The supernatant were harvested and inactivated with 0.5% methanal (48 h at 4 °C). The inactivated vaccine underwent common intestinal pathogen testing via RT-qPCR to detect potential contamination with PEDV, PDCoV, TGEV, PoRV, and PBoV. Viral inactivation was confirmed through three successive passages in Vero cell cultures. Additionally, a safety assessment was conducted by inoculating three piglets with the inactivated autogenous vaccine. Clinical parameters, including body temperature, vomiting, diarrhea, lethargy, and appetite loss, were monitored daily. The inactivated viral preparation was formulated as an autogenous vaccine for emergency use following successful quality control testing. All sows (*n* = 4,600) intramuscular received primary immunization, and with a booster immunization after 2-week. Piglet mortality rates were recorded weekly, with particular attention to pre-vaccination baseline and post-vaccination periods.

### Serological analysis

Seventeen paired serum and milk samples were collected from sows in Production Line 2 prior to vaccination. Following immunization, 15 serum and milk samples were obtained 2 weeks after the booster vaccination. Samples were centrifuged (5,000 rpm, 10 min) and stored at −20 °C until analysis.

For the serological analysis, anti-PEDV IgA and IgG antibody test kits were procured from IDEXX Laboratories, Inc. (Westbrook, Maine, USA) and Combetter Biological, Inc. (Changsha, China), respectively. All antibody tests were conducted according to the manufacturers' instructions. An S/P-value ≥0.50 was considered positive for anti-PEDV IgA, while an S/P ratio ≥0.4 indicated positivity for anti-PEDV IgG.

Neutralizing antibody titers were assessed using the microtiter method. The TCID_50_ of the PEDV XWF/2025 strain was first determined. Milk samples, along with PEDV-positive and PEDV-negative controls, were inactivated at 56 °C for 30 min, followed by two-fold serial dilutions. A volume of 50 μL of each milk dilution was mixed with 200 TCID_50_/0.1 mL of PEDV, and the mixtures were incubated at 37 °C for 1 h. After incubation, the mixtures were added to Vero cell monolayers, which were further incubated at 37 °C in a 5% CO_2_ atmosphere for 5 days. Cytopathic effects (CPE) were monitored daily and neutralizing antibody titers were calculated using the Reed-Muench method. The titer was expressed as the Log2 of the highest dilution that inhibited CPE.

### Statistical analysis

Antibody data were analyzed using GraphPad Prism 8.0 (La Jolla, CA, USA). Independent *t*-tests compared pre- and post-vaccination responses, with significance set at *p* < 0.05.

## Results

### Pathogen identification

Comprehensive diagnostic testing of intestinal samples from three infected piglets confirmed PEDV as the sole causative agent, with negative results for TGEV, PDCoV, PoRV, and PBoV (Supplementary Figure S1).

### Virus isolation and characterization

**Figure 1:**
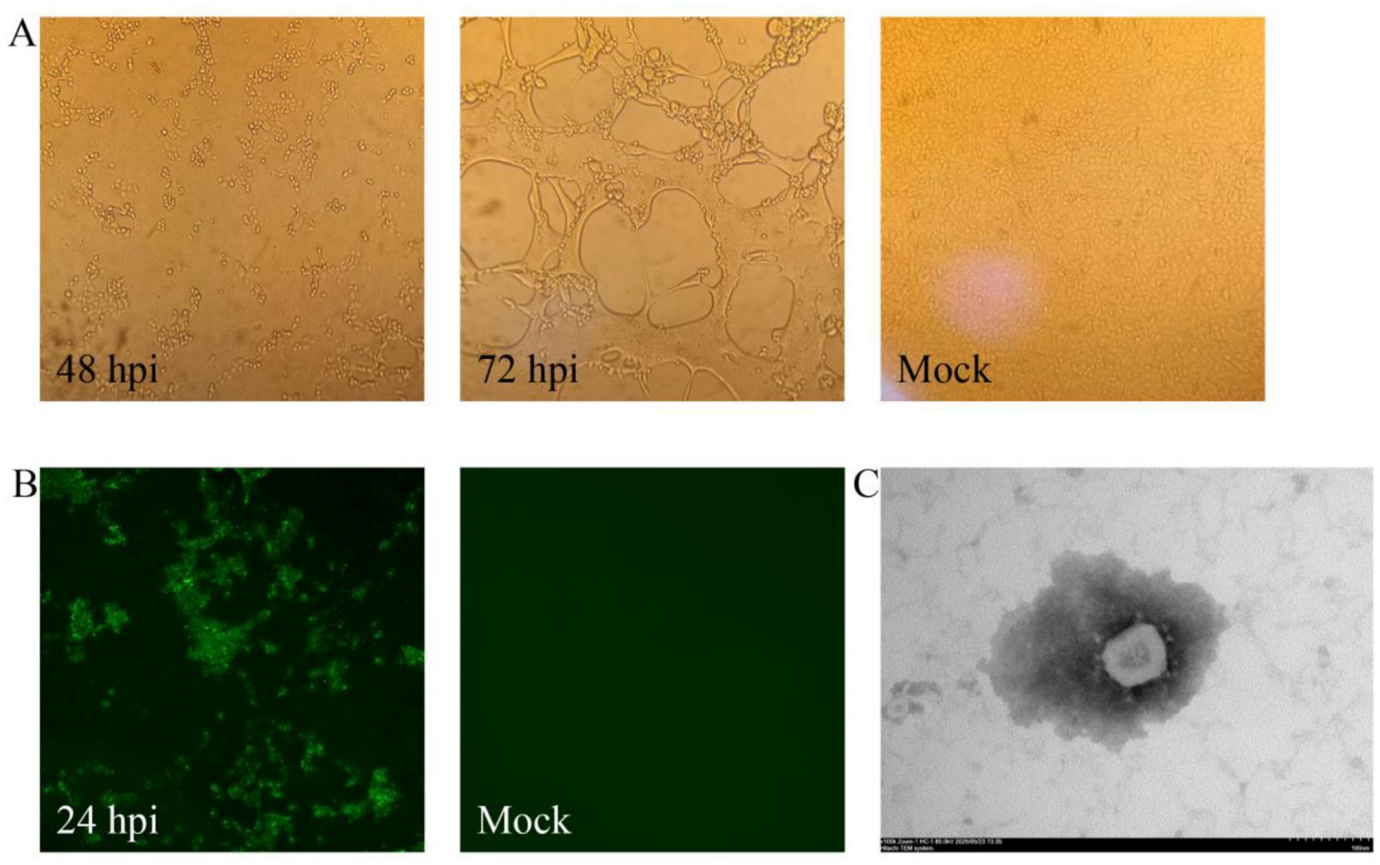
Isolation and characterization of PEDV strain XWF/2025. **(A)** Cytopathic effects (CPE) observed in Vero cells at 48 and 72 hpi with PEDV XWF/2025, compared to mock-infected control cells. Original magnification, × 200. **(B)** Immunofluorescence (IF) detection of PEDV in XWF/2025-infected Vero cells at 24 hpi and negative control (mock-infected cells). Original magnification, × 200. **(C)** Transmission electron micrograph of PEDV XWF/2025 particles showing characteristic crown-shaped spikes (inset). Samples were negatively stained with 2% phosphotungstic acid. Scale bar: 100 nm.

Following two serial passages in Vero cells, distinct cytopathic effects (CPE) became apparent, initially manifesting as syncytium formation at 48 hpi (Figure 1A). Complete CPE, characterized by cellular shrinkage and detachment, developed between 72–96 hpi (Figure 1A). The successfully isolated strain, assigned as XWF/2025, was maintained through eight consecutive passages in Vero cells.

Viral propagation was confirmed at the third passage by immunofluorescence assay (IFA) using PEDV N-specific monoclonal antibodies. Specific green fluorescence signals were detected in infected Vero cells at 24 hpi, while uninfected controls remained negative (Figure 1B). Transmission electron microscopy (TEM) examination of culture supernatants revealed typical coronavirus particles (Figure 1C), with spherical virions measuring 90–110 nm in diameter and displaying characteristic surface projections. Quantitative analysis demonstrated the viral titer of XWF/2025 was 10^5.5^ TCID_50_/mL at passage 8.

### Genomic characterization and phylogenetic analysis

A near-complete PEDV XWF/2025 genome (27,544 nt) was successfully sequenced, and then submitted to GenBank (accession number PV800119). The PEDV XWF/2025 genome containing all major coding regions, including Rep, S, ORF3, E, M, and N genes. The S gene spanned 4,161 nucleotides, encoding a 1,386-amino acid glycoprotein. Comparative sequence analysis revealed the S protein shared 90.5%−99.1% amino acid identity with reference PEDV strains, with highest similarity (99.1%) to the contemporary Chinese strain CH-HK-2021 (GenBank accession: PP785988).

**Figure 2:**
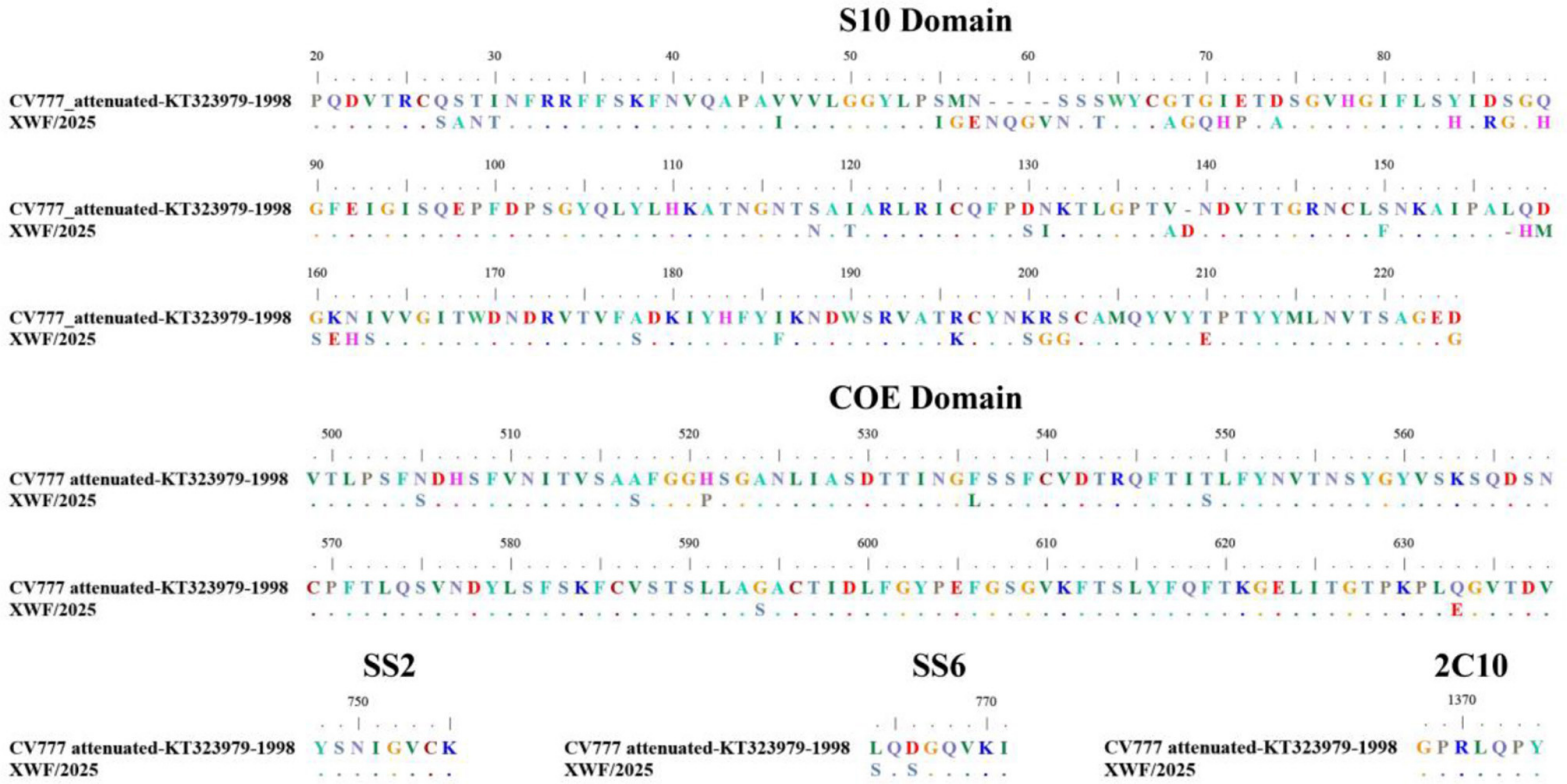
Comparative analysis of amino acid variations in neutralizing epitopes of the S protein between PEDV strain XWF/2025 and vaccine strain CV777. Sequence alignment highlights insertions, deletions, and substitutions in five major antigenic domains: S10, COE, SS2, SS6, and 2C10. The reference strain CV777 (GenBank accession no. KT323979) is shown with dots representing conserved residues. Multiple sequence alignment was performed using MEGA 6.06, and the figure was generated with BioEdit software (v7.2.5).

Comparative analysis with the reference strain CV777 revealed 111 amino acid (aa) mutations in the S protein (Supplementary Figure S2). Notably, we identified significant aa variations, including mutations, deletions, and insertions, across five critical antigenic epitopes (S10, COE, SS2, SS6, and 2C10) of the S protein (Figure 2). The XWF/2025 strain demonstrated high conservation in the SS2 and 2C10 epitopes relative to CV777. However, substantial variations were observed in epitopes of SS6 (L764S and D766S) and COE (N505S, A517S, H521P, F536L, T549S, G594S, and Q633E). In S10 domain, there were five aa insertions (58NQGV61, 139D) and 40 aa mutations distributed across multiple positions (27–30, 42, 55–57, 62, 64, 68–72, 74, 84, 86–87, 89, 118, 120, 130–131, 138, 150, 158–163, 178, 186, 196, 200–202, 210, and 224; Figure 2). These extensive modifications in key antigenic epitopes likely contribute to reduced efficacy of CV777-derived vaccines and may facilitate viral immune escape. The accumulation of mutations in these neutralizing epitopes suggests potential antigenic drift that could compromise vaccine-induced protection against circulating strains.

**Figure 3:**
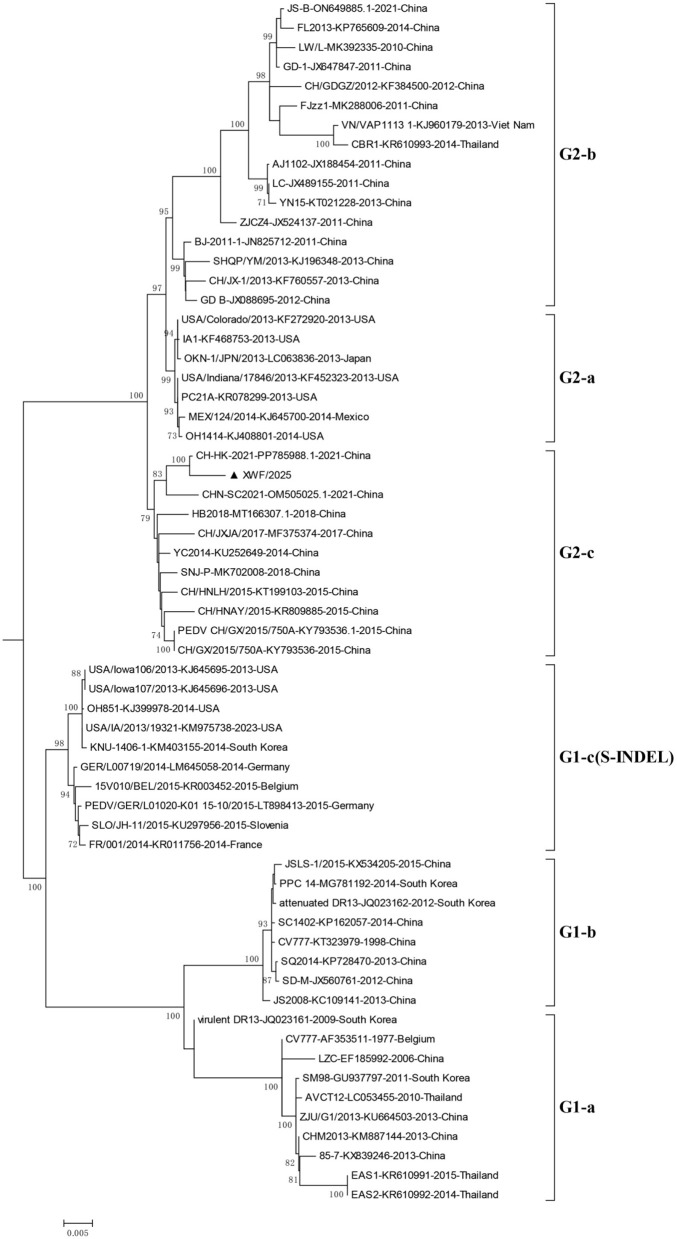
Phylogenetic analysis of PEDV strains based on complete S gene sequences. The maximum-likelihood tree was constructed using the Tamura-Nei model with gamma-distributed rates (G) in MEGA 6.06. Bootstrap values (>70%) from 1,000 replicates are shown at branch nodes. The analysis included 4,112 nucleotide positions. The XWF/2025 strain isolated in this study is highlighted with a triangle (▴). Reference strains are labeled with their GenBank accession numbers, collection time, and country of origin.

Phylogenetic analysis based on S gene sequences classified the PEDV strains into six distinct subgroups (G1-a, G1-b, G1-c, G2-a, G2-b, and G2-c). XWF/2025 clustered within subgroup G2-c, alongside closely related strains such as the Chinese isolate CH-HK-2021 and other prevalent epizootic variants (Figure 3).

### Quality test of the autogenous inactivated vaccine

Quality control testing of the autogenous inactivated vaccine confirmed the absence of common intestinal pathogens (PDCoV, TGEV, PoRV, and PBoV) (Supplementary Figure S3A), demonstrating freedom from contaminating enteric pathogens. Viral inactivation was validated through three serial passages in Vero cell cultures with no cytopathic effect observed (Supplementary Figure S3B). Safety evaluation in piglets revealed no febrile response or other clinical abnormalities following vaccination (Supplementary Figure S3C).

### Immune protective effect of the autogenous inactivated vaccine

**Figure 4:**
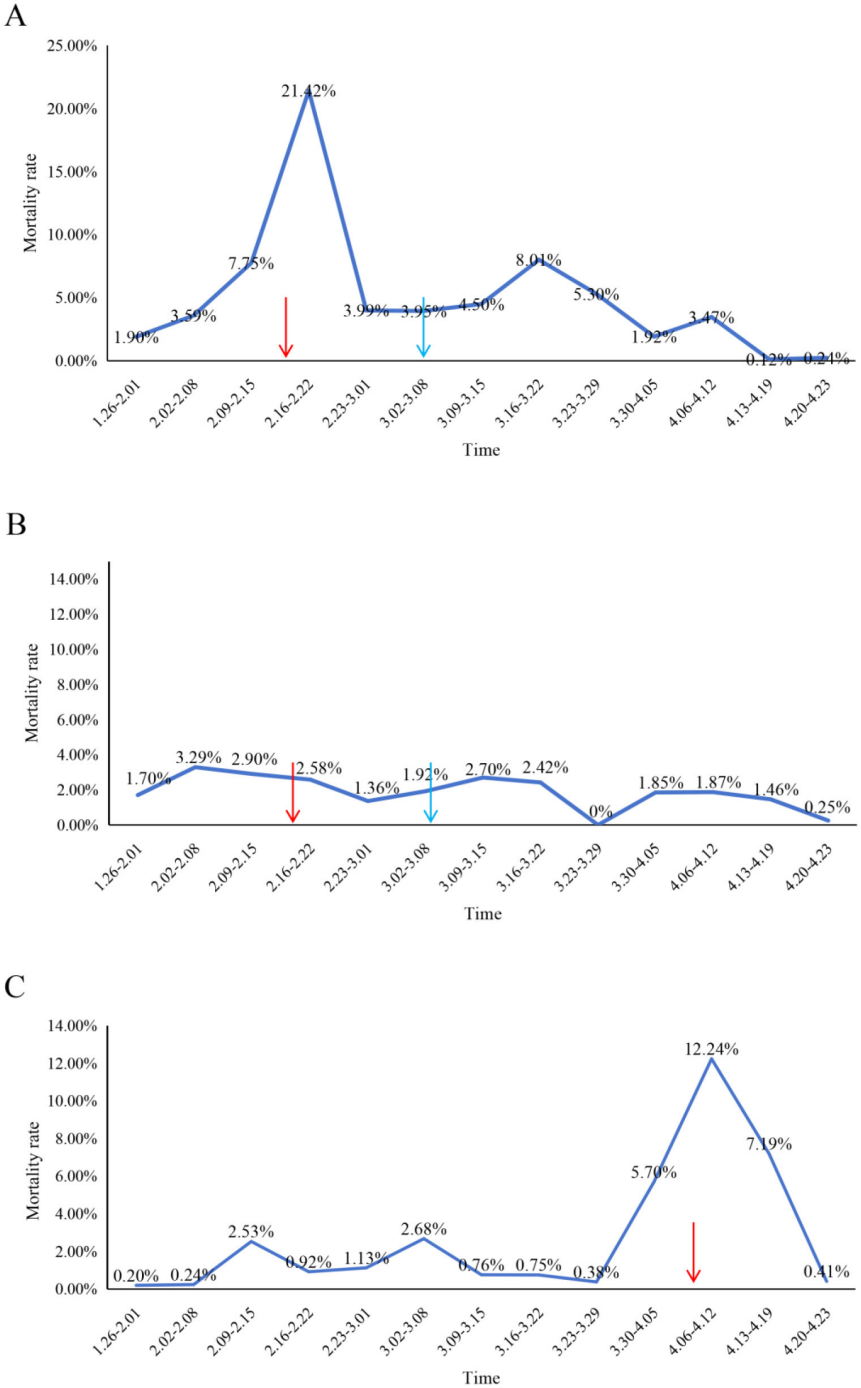
Temporal dynamics of piglet mortality rates following autogenous vaccination in affected production lines. **(A)** Production Line 2, **(B)** Production Line 3, and **(C)** Production Line 1. Mortality rates (y-axis) are plotted against time (x-axis), with red arrows indicating primary immunization and blue arrows denoting booster administration of autogenous inactivated vaccine. Production Line 1 mortality data following booster immunization were not available for complete longitudinal assessment.

After identification of PEDV as the outbreak pathogen, an autogenous inactivated vaccine derived from the tissues of infected piglet was administered to all sows in the infected unit (Production Line 2) as part of a PEDV acclimatization strategy (Figure 4A). Concurrently, Production Line 3 (uninfected) received vaccination as a preventive measure, while Production Line 1 (uninfected) remained unvaccinated to serve as a control (Figures 4B, C). Following emergency vaccination, the mortality rate of newborn piglets in the farrowing room of Production Line 2 declined significantly within 2 weeks, and production stability was gradually restored (Figure 4A).

However, Production Line 1 experienced a PED outbreak approximately 2 months later (Figure 4C). Similarly, an emergency intramuscular vaccination with the autogenous inactivated vaccine was administered to all sows in this unit. Remarkably, emergency vaccination led to a rapid reduction in newborn piglet mortality within 2 weeks, and production returned to stable levels (Figure 4C). Throughout the outbreak period in Production Line 1, both Production lines (2 and 3) previously immunized with the autogenous inactivated vaccine remained free of PED outbreaks while maintaining stable production performance (Figures 4A, B). These results demonstrate that the autogenous inactivated vaccine was effective in controlling PED.

### Immune response evaluation before and after immunization autogenous inactivated vaccine

**Figure 5:**
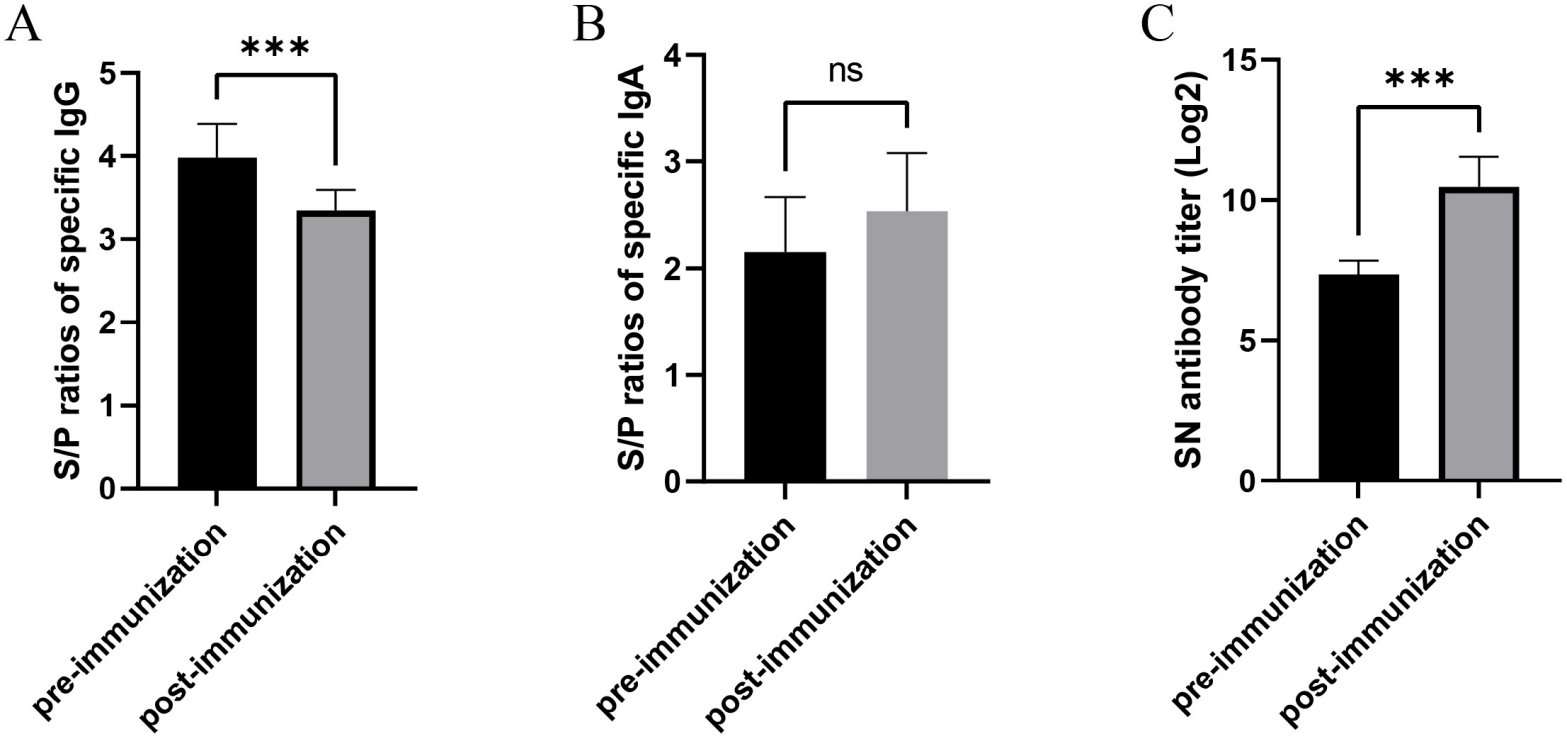
Humoral immune responses in sows following autogenous inactivated PEDV vaccination. **(A)** Serum PEDV-specific IgG levels and **(B)** milk IgA levels were quantified by ELISA, while **(C)** milk neutralizing antibody titers were determined by virus neutralization assay. All measurements were performed pre- and post-vaccination with autogenous inactivated PEDV vaccine in Production Line 2. Statistical significance was assessed by independent-samples *t*-test (ns = not significant; *p < 0.05; **p < 0.01; ***p < 0.001). Data represent mean ± SEM.

Serological analysis revealed that anti-PEDV IgG antibodies were detectable in 100% of sows in Production Line 2 both pre- and post-immunization. However, quantitative assessment showed significant decreases in antibody levels following vaccination. The mean S/P ratios for anti-PEDV IgG antibodies were 3.34 ± 0.25 after immunizations, representing a statistically significant decline compared to pre-vaccination levels (3.98 ± 0.40; Figure 5A, *p* < 0.0001).

For mucosal immunity, the seropositivity rate of anti-PEDV IgA antibodies increased from 76% pre-vaccination to 100% post-vaccination in Production Line 2 sows. The mean S/P-values for IgA antibodies showed no significantly rise following vaccination (2.54 ± 0.54), compared to baseline levels (2.15 ± 0.52; Figure 5B, *p* > 0.05).

Neutralizing antibody analysis demonstrated 100% seropositivity both before and after vaccination. However, the mean neutralizing antibody titers increased substantially post-immunization, reaching 10.47 ± 1.06 after vaccination, which was significantly higher than pre-vaccination titers (7.35 ± 0.49; Figure 5C, *p* < 0.0001).

## Discussion

Porcine epidemic diarrhea (PED) remains one of the most economically significant diseases affecting the global swine industry. In China, PED exhibits a year-round sporadic prevalence, with disease control primarily relying on biosecurity measures, vaccination, and therapeutic interventions (1). Given that PED predominantly causes high mortality in neonatal piglets, vaccination strategies focus on immunizing sows to confer passive lactogenic immunity via colostrum and milk (18). However, the high mutation rate of PEDV often results in poor cross-protection of commercial vaccines against circulating field strains, contributing to recurrent outbreaks (19). Consequently, many farms resort to feedback exposure (intentional infection of sows with field virus) to mitigate losses, despite the inherent risks of viral dissemination and potential contamination with exogenous pathogens (20). In this study, we successfully controlled a PED outbreak by rapidly identifying the causative strain, inactivating its infected tissues, and administering it as an emergency autogenous vaccine. Clinical symptoms subsided significantly within 2 weeks post-vaccination, demonstrating the efficacy of this approach. Our protocol ensured both antigenic matching with the circulating strain and vaccine safety, suggesting its potential as a scalable and effective PED control strategy. However, due to the emergency context of the outbreak, it was not practically feasible to include an unvaccinated control group. This limitation precludes definitive conclusions regarding vaccine efficacy through direct comparative analysis. Future studies under controlled conditions will be necessary to validate these findings and further establish the protective effectiveness of this autogenous vaccine approach.

The rapid containment of the outbreak was further facilitated by stringent biosecurity measures. Strict segregation of personnel and materials within affected units, with thorough disinfection of equipment after use (21). Enhanced disinfection protocols, including chlorine-based treatment of farrowing crates followed by drying powders post-diarrhea episodes. Routine fogging disinfection (thrice daily) of farrowing aisles using 1:500 perchloride solutions. Water sanitation, with chlorine-based disinfectants or acidifiers added to drinking water to inactivate potential viral contaminants (22). Early intervention, including immediate euthanasia of severely affected neonatal piglets (< 7 days) exhibiting watery diarrhea, followed by crate drying (23). Supportive care for older piglets (>7 days), including oral rehydration therapy (electrolytes and milk replacers) combined with antibiotics (gentamicin and amoxicillin) and early weaning when feasible.

Prior to 2010, G1-genotype PEDV strains predominated in China, typically causing mild clinical signs (24). However, post-2010, highly pathogenic G2 variants emerged, leading to piglet mortality rates of 80%−100% and nationwide spread (25, 26). Between 2017 and 2020, G2-b subgenotypes became dominant, while recent surveillance indicates an increasing prevalence of G2-c strains (3, 9, 27). Novel recombinant variants (S-INDEL-like) have also been detected in some regions, likely arising from recombination between field and vaccine strains (24, 28). In this study, genetic analysis classified the outbreak strain as G2-c, sharing 96.5% whole-genome homology and 93.1% S gene homology with the CV777 vaccine strain. Our genomic analysis identified several critical mutations in the S protein of the XWF/2025 strain, located within key neutralizing epitopes (S10, COE, and SS6). Among these, the mutation at position 766 within the SS6 epitope has been shown to contribute to pathogenic evolution under intense immune pressure and to facilitate immune escape (15). Notably, recent reports of highly virulent PEDV strains circulating in China have documented similar mutational patterns (27, 29). The rapid accumulation of mutations in these neutralizing epitopes highlights the significant evolutionary pressure imposed by widespread vaccination. This phenomenon helps explain the limited cross-protection offered by CV777-based vaccines against emerging variants and underscores the urgent need for updated vaccines matched to currently circulating strains.

The S protein exhibited multiple mutations, including in known antigenic epitopes, explaining the limited cross-protection of CV777-based vaccines against contemporary G2 strains. Notably, while autogenous vaccination significantly elevated PEDV-specific neutralizing antibodies, the pre-existing high antibody levels in most sows prior to vaccination further underscored the suboptimal efficacy of current vaccines. These findings highlight the urgent need for variant-matched vaccines tailored to prevalent PEDV strains.

Furthermore, our study revealed that autogenous immunization did not significantly increase anti-PEDV IgA antibodies in sow milk, while serum anti-PEDV IgG antibodies unexpectedly decreased. These findings suggest that relying solely on IgG and IgA antibody levels may be an unreliable method for evaluating the protective efficacy of PEDV vaccines. Recent research has demonstrated that neutralizing antibody titers serve as the critical determinant in protecting newborn piglets against PEDV infection (30). Specifically, when sows achieve serum neutralizing titers ≥1:377 at 1 week pre-farrowing, their offspring exhibit significantly improved survival rates (>80%) following PEDV exposure (30). Current commercial inactivated PEDV vaccines in China show limited efficacy against emerging G2-c genotype strains. While these vaccines can induce substantial IgG antibody responses, their neutralizing capacity against G2c variants remains significantly inferior to that of wild-strain-derived inactivated vaccines (30). This discrepancy highlights the importance of prioritizing neutralizing antibody assessment over conventional antibody measurements when evaluating vaccine efficacy. Consequently, we propose that future PEDV vaccine development and evaluation should emphasize neutralizing antibody titers as the primary correlate of protection.

## Conclusion

In conclusion, the PEDV landscape in China is characterized by continuous viral evolution, increasing recombination events, and growing control challenges. To address this, future efforts must prioritize enhanced molecular surveillance to track emerging strains, rational vaccine design targeting dominant variants, and integrated biosecurity and immunization protocols. This study demonstrates the effectiveness of a rapid response strategy combining autogenous vaccination and precision biosecurity, providing a viable model for PED mitigation in the face of viral diversity.

## Data Availability

The data presented in this study are available within the article. Raw data supporting this study are available from the corresponding authors.

## Supplementary material

Supplementary Figure S1 Detection of enteric pathogens in intestinal samples from infected piglets. Representative RT-qPCR detection of **(A)** PEDV, **(B)** TGEV, **(C)** PDCoV, **(D)** PoRV, and **(E)** PBoV were performed in intestinal samples from three affected piglets. PC: positive control.

Supplementary Figure S2 Comparative analysis of S protein sequences between PEDV strain XWF/2025 and vaccine strain CV777. Complete S protein amino acid alignment showing sequence variations relative to the reference strain CV777 (GenBank: KT323979). Dots indicate conserved residues. Multiple sequence alignment was performed using MEGA 6.06 with ClustalW algorithm, and the visualization was generated using BioEdit 7.2.5.

Supplementary Figure S3 Quality control assessment of the autogenous inactivated vaccine. **(A)** RT-qPCR analysis for contaminating enteric pathogens (PDCoV, TGEV, PoRV, and PBoV) in the vaccine preparation. **(B)** Third-passage Vero cell cultures following incubation with the inactivated vaccine, showing absence of cytopathic effects. **(C)** Rectal temperature monitoring of piglets following intramuscular administration of the vaccine.
