# Supplementary figures and images for: Effective control of the emerging PEDV G2-c variant with an inactivated autogenous vaccine
Source: Front Vet Sci. 2025 Oct 14;12:1697499. doi: 10.3389/fvets.2025.1697499 (PMC12559854; doi:10.3389/fvets.2025.1697499)

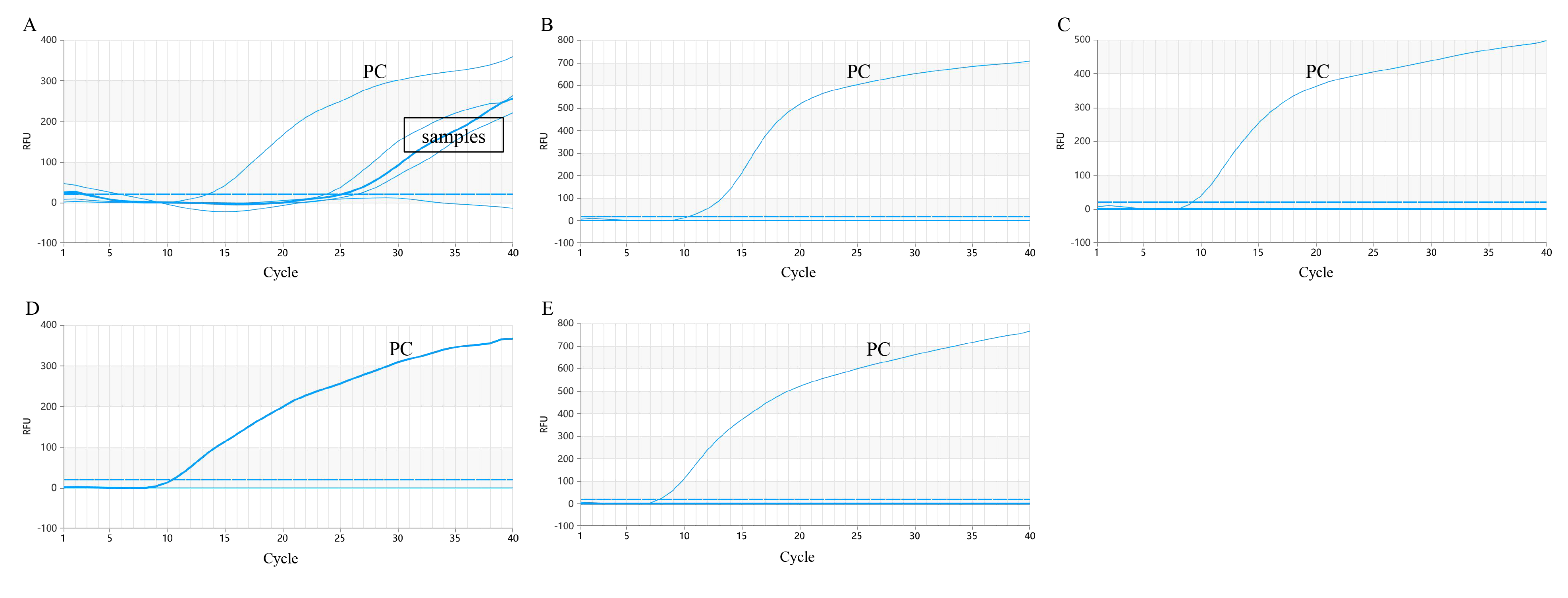

Supplement: Supplementary Figure S1 — Detection of enteric pathogens in intestinal samples from infected piglets. Representative RT-qPCR detection of (A) PEDV, (B) TGEV, (C) PDCoV, (D) PoRV, and (E) PBoV were performed in intestinal samples from three affected piglets. PC: positive control. [file Image_1.png]

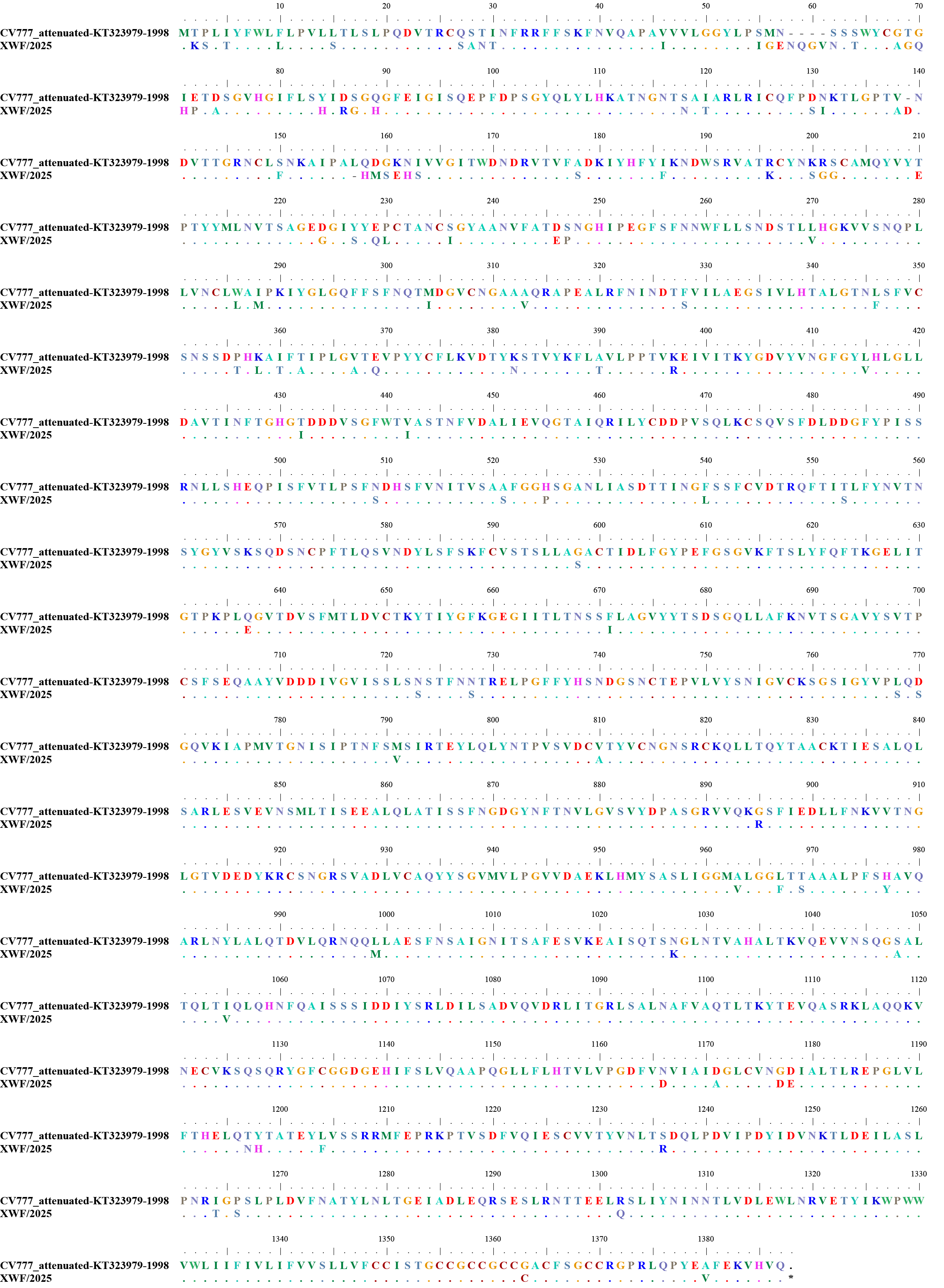

Supplement: Supplementary Figure S2 — Comparative analysis of S protein sequences between PEDV strain XWF/2025 and vaccine strain CV777. Complete S protein amino acid alignment showing sequence variations relative to the reference strain CV777 (GenBank: KT323979). Dots indicate conserved residues. Multiple sequence alignment was performed using MEGA 6.06 with ClustalW algorithm, and the visualization was generated using BioEdit 7.2.5. [file Image_2.png]

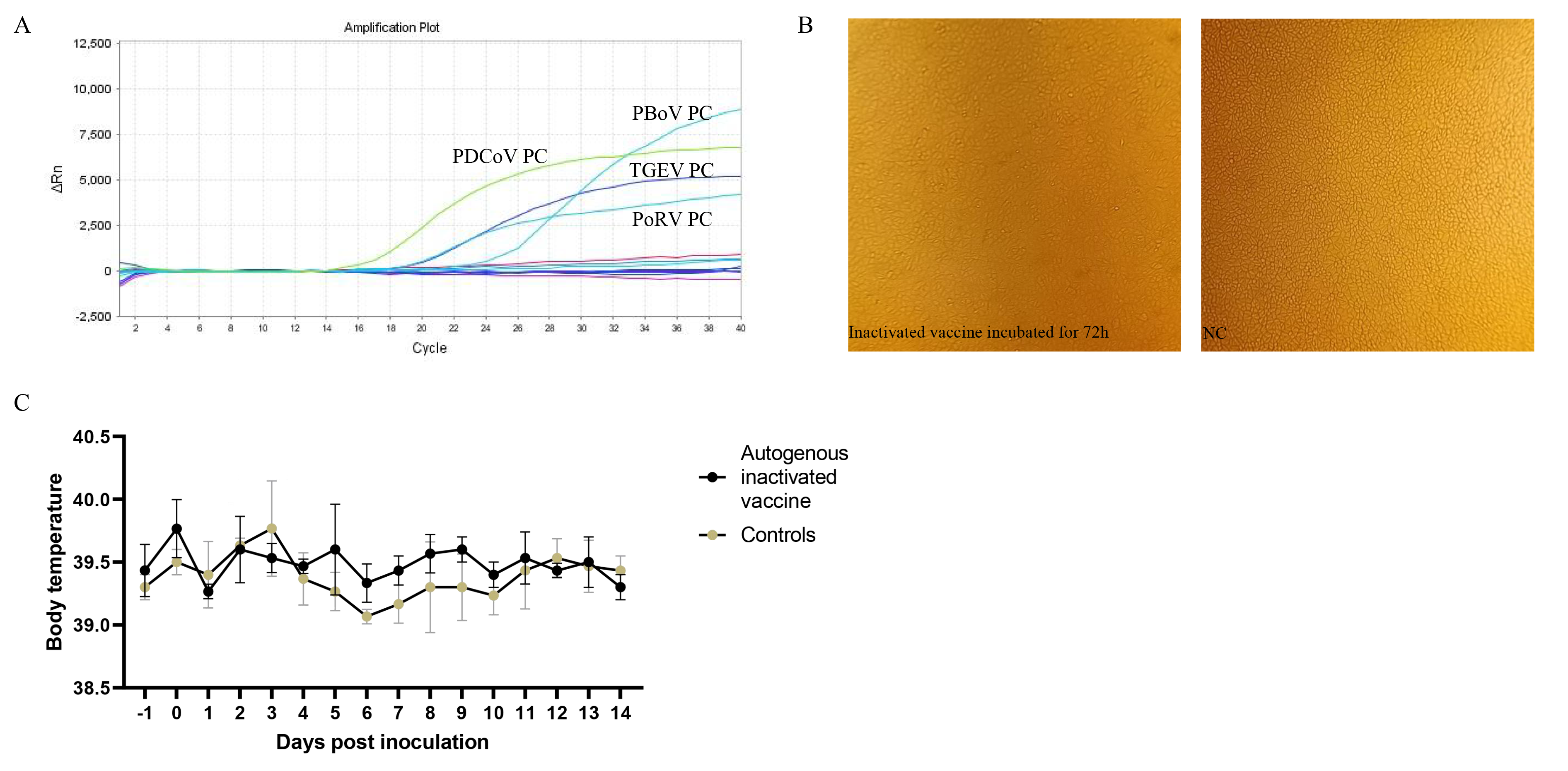

Supplement: Supplementary Figure S3 — Quality control assessment of the autogenous inactivated vaccine. (A) RT-qPCR analysis for contaminating enteric pathogens (PDCoV, TGEV, PoRV, and PBoV) in the vaccine preparation. (B) Third-passage Vero cell cultures following incubation with the inactivated vaccine, showing absence of cytopathic effects. (C) Rectal temperature monitoring of piglets following intramuscular administration of the vaccine. [file Image_3.png]
